# Regulation of the Inflammatory Response, Proliferation, Migration, and Epithelial-Mesenchymal Transition of Human Lens Epithelial Cells by the lncRNA-MALAT1/miR-26a-5p/TET1 Signaling Axis

**DOI:** 10.1155/2023/9942880

**Published:** 2023-01-16

**Authors:** Yaru Hu, Xue Han, Yue Chen, Jinbiao Cai, Juan Li, Yuchen Fan, Jianfeng Wang, Shanglun Xie

**Affiliations:** ^1^Department of Ophthalmology, The First Affiliated Hospital of Bengbu Medical College, Bengbu, Anhui 233004, China; ^2^School of Life Sciences, Anhui Province Key Laboratory of Translational Cancer Research, Bengbu Medical College, Bengbu, Anhui 233030, China

## Abstract

**Background:**

The ocular inflammatory microenvironment has been reported to be closely associated with the occurrence and progression of highly myopic cataract (HMC). Long noncoding RNA metastasis-associated lung adenocarcinoma transcript 1 (MALAT1) could alter the biological properties of mammalian cells by modulating the expression of inflammatory mediators; therefore, it may contribute to the development of HMC.

**Objective:**

To investigate the function of MALAT1 in the inflammatory response, proliferation, migration, and epithelial-mesenchymal transition (EMT) of inflammatory and injured human lens epithelial cells (HLECs) and to reveal the underlying molecular signals.

**Methods:**

Patients with HMC and age-related cataract (ARC) with an axial length of more than 26 mm were selected, and the anterior capsular tissue was obtained during cataract surgery. TNF-*α* (20 ng/mL) was chosen to induce inflammatory damage in HLECs to simulate the inflammatory microenvironment in HMC eyes. Specific siRNAs, inhibitors, and mimics were used to suppress or enhance the functions of MALAT1 and miR-26a-5p. RT-qPCR and Western blot analysis were performed to measure gene and protein expression, respectively.

**Results:**

The expression of MALAT1 and the inflammatory mediators IL-6, MMP-2, and MMP-9 were significantly higher in HMC anterior capsule tissues than in ARC. TNF-*α* treatment increased the expression of MALAT1, while it also promoted the proliferation, migration, and EMT of HLECs. MALAT1 interference decreased the expression of IL-6 and MMP-2 and inhibited the aforementioned processes. Furthermore, MALAT1 negatively regulated the expression of miR-26a-5p and then promoted TET1 expression. TET1 was identified as a direct target of miR-26a-5p, and the promoting effect of MALAT1 on TET1 expression could be reversed by miR-26a-5p mimics.

**Conclusion:**

The inflammatory environment and MALAT1 expression could be reciprocally induced in HLECs. MALAT1 may act as a ceRNA via the “sponge” miR-26a-5p and target TET1 to regulate the inflammatory response, proliferation, migration, and EMT processes in HLECs.

## 1. Introduction

High myopia, a condition characterised by an axial length of ≥26 mm or a dioptre number greater than −6.0 D, is the leading cause of visual impairment in people aged 45 to 59 in China [1]. As a relatively distinct cataract phenotype, highly myopic cataracts (HMCs) exhibit early onset and rapid progression, as well as a high risk of blindness that is often accompanied by posterior staphyloma, choroidal retinal atrophy, scleral thinning, and visual papillary deformation [2]. The incidence of posterior capsular cataracts after surgical operations is higher than that of age-related cataracts (ARCs). Other serious complications, such as capsular constriction and dislocation of the artificial lens capsule complex, occur more readily.

Recent studies have shown that the concentrations of inflammatory mediators, such as MMP-2, IL-1, MCP-1, tumour necrosis factor-*α* (TNF-*α*), and NF-KB, in the aqueous humour of HMC patients are higher than those of ARC patients [3, 4]. Thus, the intraocular inflammatory environment may be associated with an early onset, a severe postoperative inflammatory response, and a high incidence of complications.

Long noncoding RNAs (lncRNAs) have been recognised to play a crucial role in epigenetic modification as well as transcriptional and posttranscriptional regulation, which further regulates cell proliferation, apoptosis, activity, oxidative stress, immunisation, and inflammatory responses [5]. Metastasis-associated lung adenocarcinoma transcript 1 (MALAT1), one of the earliest identified lncRNAs, is expressed in many mammalian cells. MALAT1 is an approximately 8.7 kb intergenic transcript located on chromosome 11q13. Although the main transcript has been processed into 6.7 kB, it cannot be translated into proteins owing to the lack of an open-coding framework. MALAT1 plays an important regulatory role in the tumour inflammatory environment and increases the proliferation, migration, and epithelial-mesenchymal transition (EMT) process of tumour cells [6–8]. The increased expression of MALAT1 can trigger cell inflammatory responses by increasing the expression of inflammatory mediators IL-6 and TNF-*α*, whereas MALAT1 interference has the opposite effect [9–11]. High levels of MALAT1 enhance retinal inflammation in diabetic retinopathy [12], suggesting that MALAT1 is likely also involved in regulating the inflammatory response of ocular diseases. However, the role of MALAT1 in the regulation of the inflammatory microenvironment in HMC eyes has not been well studied, nor has its contributions to clinical symptoms and postoperative complications for HMC patients.

Recently, a novel competitive endogenous ribonucleic acid (ceRNA) regulatory mechanism has been proposed. When ceRNAs (similar to lncRNAs and mRNAs) have the same miRNA response element (MRE), the MRE is competitively shared to mediate the posttranscriptional regulation of lncRNAs and mRNAs [8, 13]. MALAT1 is localised to the cytoplasm of human lens epithelial cells (HLECs) [14]. Therefore, it may also function as a ceRNA for some miRNAs and mRNAs as well as upregulate the expression of inflammatory mediator genes at the posttranscriptional level. Investigating the molecular functions of MALAT1 for regulating the inflammatory response, proliferation, migration, and EMT of HLECs in an inflammatory state will help elucidate the pathological mechanism of HMCs and lead to the development of novel effective treatments.

In this study, we showed that the expression levels of inflammatory mediators and MALAT1 were observably increased in HMC patients compared with those in ARC patients. *In vitro*, TNF-*α* treatment increased the expression of MALAT1 and promoted the proliferation, migration, and EMT of HLECs, whereas MALAT1 interference suppressed both the inflammatory response and the aforementioned processes. Furthermore, MALAT1 silence raised the expression of miR-26a-5p but decreased the TET1 level. miR-26a-5p directly binds to TET1. miR-26a-5p mimics reduced the expression of MALAT1 and TET1, whereas inhibitors have the opposite effect, suggesting that MALAT1 may function as a ceRNA for regulating miR-26a-5p and TET1 to modulate the inflammatory microenvironment and biological behaviour of HLECs. Overall, our results showed that the expression of MALAT1 and inflammatory mediators could be mutually induced and that the MALAT1/miR-26a-5p/TET1 signalling axis was recruited in this process.

## 2. Materials and Methods

### 2.1. Ethics Statement

HMC and ARC anterior capsular tissues were obtained from the Ophthalmology Department of the First Affiliated Hospital of Bengbu Medical College. According to the principles outlined in the Declaration of Helsinki, tissue specimens and written informed consent signed by the patient or his/her family members were obtained from each patient during cataract surgery, as approved by the Ethics Review Committee of the Scientific Research Department of the First Affiliated Hospital of Bengbu Medical College (Approval Number: 2022328).

### 2.2. Collection of Lens Anterior Capsule Tissues

Clinical specimens were obtained from 36 HMC patients and 36 ARC patients. The patients ranged from 46 to 79 years in age. The inclusion criteria were as follows: (1) HMC patients: ocular axis >26 mm, myopia degree >6.00 D; (2) ARC patients: nuclear (HMCs are mostly nuclear in type), age <80 years old (matching the ages of the HMC patients). The exclusion criteria were as follows: (1) patients with systemic diseases such as diabetes and immune system diseases that may cause inflammatory reactions; (2) patients with ocular diseases such as uveitis, glaucoma, and retinal choroiditis that may cause intraocular inflammatory reactions. Anterior capsule tissues (including lens epithelial cells) with a diameter of 5.5–6.0 mm in the centre of the lens were torn off during the operation and immediately stored in a freezer at −80°C.

### 2.3. Detection of Inflammatory Factors and TET1 Expression in Anterior Capsule Tissues

Total RNA and protein were extracted from the anterior capsule tissues of HMC and ARC patients by tissue milling. RT-qPCR and Western blotting experiments were conducted to detect the expression of inflammatory factors TNF-*α*, IL-6, MMP-2, and TET1 at the mRNA or protein level.

### 2.4. Cell Culturing and Establishment of an Inflammatory Injury Cell Model

HLECs (HLEC-SR01/04) were purchased from Saiku (Guangzhou, China). HLECs were cultured in Dulbecco's Modified Eagle's Medium (DMEM) containing 15% foetal bovine serum (FBS; Gibco, Billings, MT, USA) and 1% penicillin-streptomycin (Thermo Scientific, Waltham, MA, USA) in a 5% CO_2_ atmosphere at 37°C. TNF-*α* (PeproTech, Rocky Hill, NJ, USA) was dissolved in phosphate buffer containing 0.1% bovine serum albumin and added to the culture medium at a final concentration of 20 *μ*g/L to induce an HLEC inflammatory response for 48 h.

### 2.5. Cell Transfection

Small interfering RNAs targeting MALAT1 (siRNA-1 and siRNA-2) as well as hsa-miR-26a-5p mimics and inhibitors, which were purchased from Bioengineering (Shanghai, China), were transfected into HLECs using Lipofectamine 2000 (Invitrogen, Carlsbad, CA, USA). The sequence information is shown in Supplementary Tables S1 and S2. HLECs in the logarithmic growth phase were preinoculated in 6 cm dishes for 24 h until cell fusion reached approximately 60–70%.

### 2.6. Real-Time Quantitative PCR and Western Blotting

The primer sequences used in RT-qPCR and the antibodies used for Western blotting are listed in Supplementary Tables S3 and S4. Glyceraldehyde 3-phosphate dehydrogenase (GAPDH) served as the normalisation control for RT-qPCR and Western blotting.

### 2.7. Cell Proliferation Assay

HLECs in the logarithmic phase were inoculated into 96-well plates at a density of 0.5 × 10^4^ cells per well and cultured for 24 h in a 5% CO_2_ atmosphere at 37°C. After adding 10 *μ*L of CCK-8 solution, the HLECs were incubated for a further 4 h. The absorbance at 450 nm was measured using an M100 multifunctional microplate analyser (Tecan, Männedorf, Switzerland) to evaluate the cell proliferation ability.

### 2.8. Cell Migration and Invasion Assays

Two thousand HLECs treated with TNF-*α*, MALAT1 siRNA, or negative control siRNA were inoculated on the upper surface of a 6.5 mm transwell chamber (BD Biosciences, San Jose, CA, USA), while the lower surface of the chamber was filled with 600 *μ*L of medium containing 10% FBS. After 24 h of incubation, the chamber was fixed in 4% formaldehyde for 30 min and then stained with crystal violet for 15 min. The penetrating cells were observed under an Olympus CKX53 inverted fluorescence microscope and counted in three random fields.

### 2.9. Cell Wound Healing Assay

HLECs treated with TNF-*α*, MALAT1 siRNA, and negative control siRNA were scratched when the convergence reached approximately 70%. Photographs were taken between 0 and 24 h after the cell injury using an Olympus CKX53 inverted fluorescence microscope.

### 2.10. Flow Cytometry

The cells in the logarithmic growth phase were counted and planted into six-well plates. The cells in each group were treated when the cell density reached 70–80%. After 24 or 48 h, flow samples were prepared according to the instructions of the apoptosis kit. After incubation without light for 1 h (a 300-mesh nylon net was filtered into the flow tube before feeding), a CytoFlex flow cytometer (Beckman Coulter, Indianapolis, IN, USA) was used to detect apoptosis.

### 2.11. Luciferase Assay

Detailed protocols were followed, as reported previously [15]. Wild-type and mutant binding sequences of miR-26a-5p on TET1 3′-UTR (1463–1469) were constructed into pGL3 luciferase reporter vectors and cotransfected into HLECs with miR-26a-5p mimics or their negative controls using Lipofectamine 2000. The luciferase activity was determined using a dual luciferase reporter assay system (Promega, Madison, WI, USA).

### 2.12. Bioinformatics Predictive Analysis

Online software StarBase v2.0 (https://starbase.sysu.edu.cn) and TargetScan v7.2 (https://www.targetscan.org) were used to predict the miRNAs that bind to MALAT1. miRDB (https://www.mirdb.org/) was used to search for mRNAs that serve as potential targets of miR-26a-5p.

### 2.13. Statistical Analysis

Data were obtained from ≥3 independent experiments and analysed using GraphPad Prism 8 software (La Jolla, CA, USA). For variables with a normal distribution, a two-tailed *t*-test was used to determine the significance of differences. *P* > 0.05 was considered statistically insignificant, and the following signs were used: ^*∗∗∗∗*^*P* < 0.0001, ^*∗∗∗*^*P* < 0.001, ^*∗∗*^*P* < 0.01, and ^*∗*^*P* < 0.05.

## 3. Results

### 3.1. Expression of Inflammatory Factors Increases Significantly in the Anterior Capsular Tissue of HMC Patients

**Figure 1:**
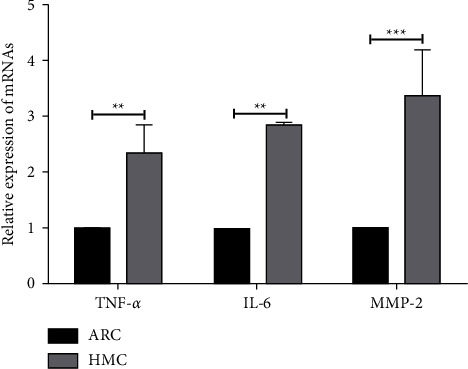
Comparison of the expressions of inflammatory factors TNF-*α*, IL-6, and MMP-2 in the anterior capsule tissues of HMC and ARC patients, as evaluated by RT-qPCR.

To investigate the pathogenesis of inflammatory stress in HMCs, we analysed the expression of inflammatory mediators in the anterior capsular tissues of HMC and ARC patients using RT-qPCR experiments. We found that the mRNA expressions of TNF-*α*, IL-6, and MMP-2 were significantly higher in the HMC tissues than in the ARC tissues (Figure 1), suggesting that the anterior capsule of HMC patients was in an inflammatory state.

### 3.2. Expression of MALAT1 Increases Markedly in the Anterior Capsular Tissue of HMC Patients

**Figure 2:**
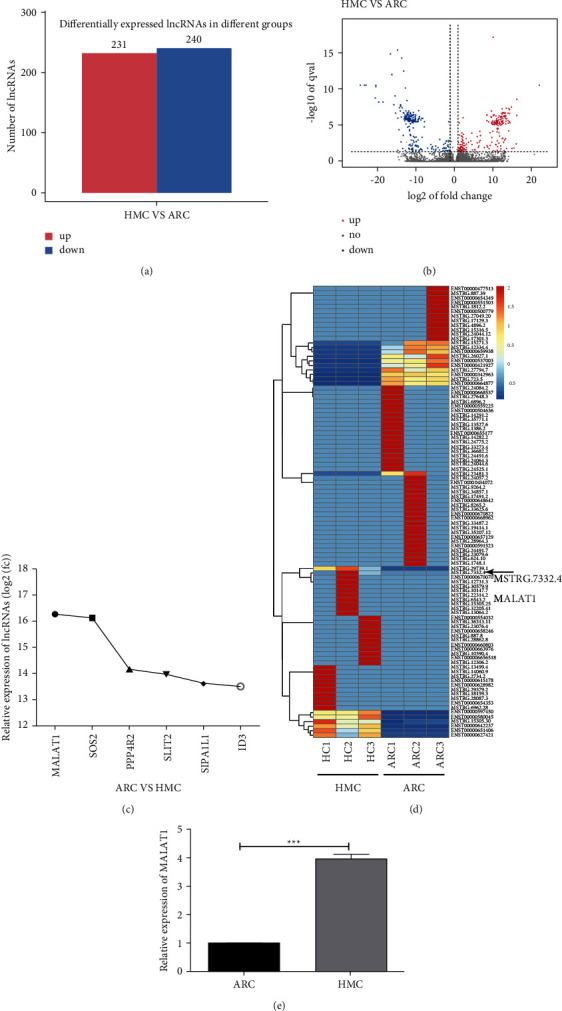
Differentially expressed lncRNAs are found in the anterior capsular tissues of HMC and ARC patients. (a) The total number of increased and decreased lncRNAs detected in HMC samples compared with the ARC group. (b) Volcano plots filtering differentially upregulated and downregulated lncRNAs with statistical significance (log2 fold change ≥1.5, *P* < 0.05). (c) Expressions of MALAT1, SOS2, SLIT2, ID3, PPP4R2, and SIPA1L1 were most increased. (d) The clustered heatmap showing MALAT1 expression was significantly higher in HMC samples than in the ARC group. (e) RT-qPCR detected MALAT1 expression that was markedly higher in HMC samples. GAPDH served as the endogenous control.

To investigate the role of lncRNA in the intraocular inflammatory stress of HMC patients, 18 anterior capsular tissue samples from HMC and ARC patients were collected during surgery. The samples were prepared for whole transcriptome sequencing to examine the expression patterns of lncRNAs in HMC anterior capsular tissues. A total of 571 lncRNAs were differentially expressed in HMC tissues compared with ARC tissues (fold change ≥1.5, *P* ≤ 0.05) (Figure 2(a)). Of these differentially expressed lncRNAs, 231 were upregulated and the remaining were downregulated (Figures 2(a) and 2(b)). In addition, the expressions of MALAT1, SOS2, SLIT2, ID3, PPP4R2, and SIPA1L1 were the most significantly altered. Notably, the MALAT1 level was upregulated more than 16 times (Figures 2(c) and 2(d)). Therefore, MALAT1 was selected, and its molecular function in inflammatory modulation was investigated in this study. We then confirmed the changes in MALAT1 expression using RT-qPCR (Figure 2(e)). Collectively, these results demonstrate that the expression of lncRNA-MALAT1 was higher in the anterior capsular tissues of HMCs than those of ARCs.

### 3.3. Expression of MALAT1 Increases in TNF-*α*-Induced Inflammatory HLECs

**Figure 3:**
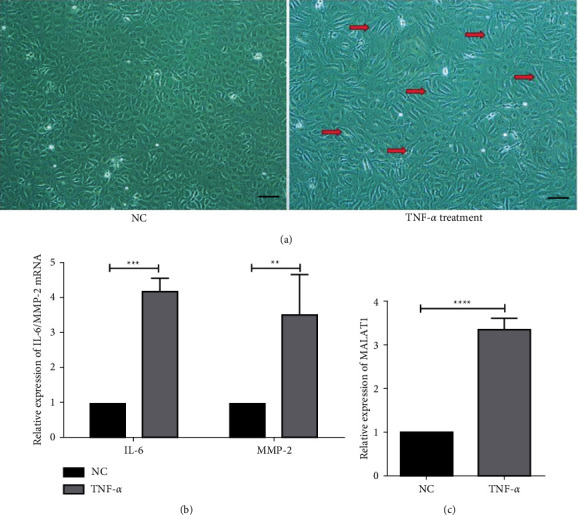
TNF-*α* treatment induces inflammatory injured HLECs. (a) Morphological changes of HLECs after TNF-*α* addition (scale: 200 *μ*m). Inflammatory HLECs were transformed into long spindle cells (arrows). (b, c) Evaluation of the changes in inflammatory factor and MALAT1 expressions by RT-qPCR in TNF-*α* treated HLECs.

**Figure 4:**
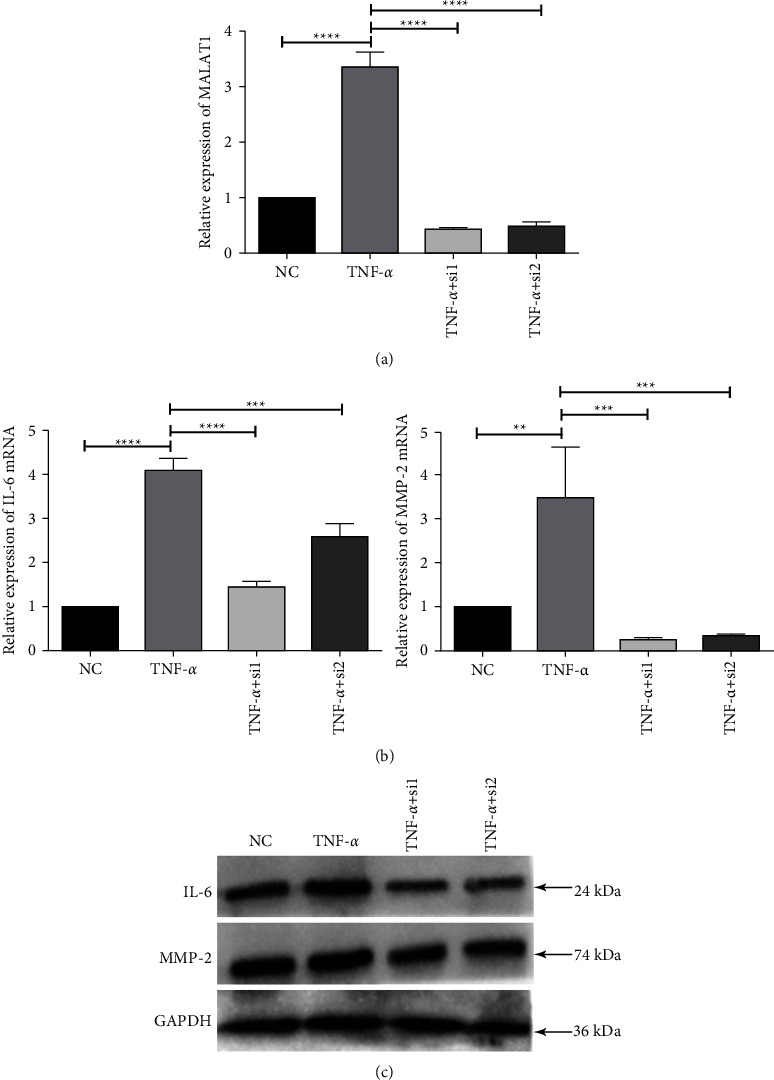
Efficiency of MALAT1 interference on the expression of inflammatory factors in TNF-*α*-treated HLECs. (a) Evaluation of the interference effectiveness of siRNAs on MALAT1 expression by RT-qPCR. (b, c) Evaluation of mRNA and protein expressions of inflammatory factors by RT-qPCR and Western blotting experiments. GAPDH served as the endogenous control.

To reveal the regulation effect of MALAT1 on the inflammatory state, we treated HLECs with TNF-*α* (20 ng/mL), with reference to previous studies, to construct an inflammatory injury cell model *in vitro*. After treatment for 48 h, the HLECs exhibited significant morphological changes from short spindle-shaped or polygonal HLECs to long spindle-shaped inflammation-damaged cells (Figure 3(a)), resembling the characteristics of fibrocytes [16]. Compared with the untreated HLECs, the mRNA and protein expressions of inflammatory factors IL-6 and MMP-2 were significantly increased, suggesting that TNF-*α* treatment causes inflammatory injury to HLECs (Figures 3(b) and 4(c)). Importantly, the expression of MALAT1 was significantly increased in the TNF-*α*-treated sample (Figure 3(c)), which is consistent with the high expression found in HMC anterior capsular tissues.

### 3.4. MALAT1 Interference Modulates the Inflammatory Response of HLECs

We interfered with MALAT1 expression using siRNAs to determine whether MALAT1 modulates the inflammatory response of HLECs. We designed two specific siRNAs for MALAT1 and transfected them into HLECs. Both siRNAs were found to significantly silence the expression of MALAT1 (Figure 4(a)). In addition, MALAT1 interference obviously decreased the mRNA and protein expressions of IL-6 and MMP-2 (Figures 4(b) and 4(c)), the concentrations of which were elevated in the TNF-*α*-treated sample. These data suggest that MALAT1 interference could modulate the inflammatory state by reducing the expression of inflammatory mediators and further that the expression of MALAT1 and inflammatory mediators could be mutually induced in HLECs.

### 3.5. MALAT1 Interference Suppresses the Proliferation, Migration, and EMT of Inflammatory HLECs

**Figure 5:**
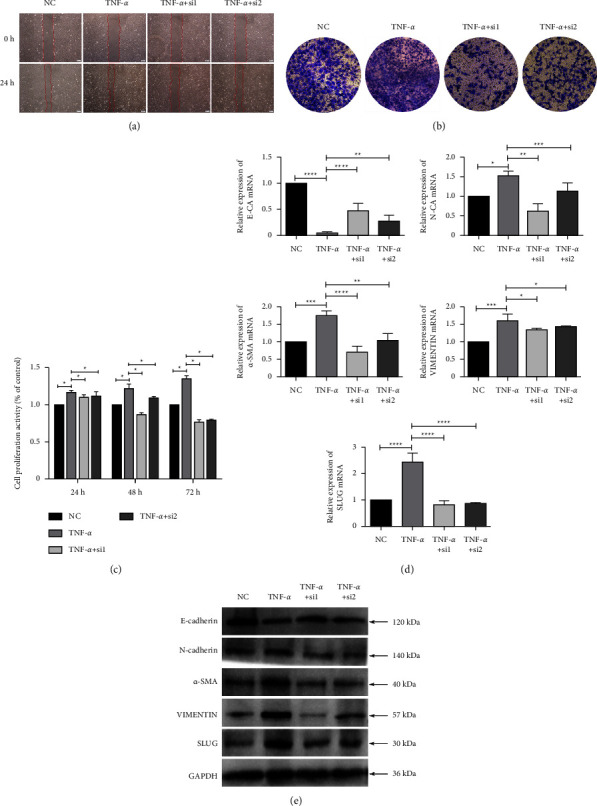
Efficiency of MALAT1 interference on proliferation, migration, and EMT of HLECs under inflammatory conditions. (a, b) Evaluation of the effectiveness of TNF-*α* treatment and MALAT1 siRNAs on the migratory ability of HLECs by wound healing and transwell assays. (c) Evaluation of the effectiveness of TNF-*α* treatment and siRNAs on the proliferation ability of HLECs by the CCK8 assay. (d, e) Evaluation of the effectiveness of TNF-*α* treatment and siRNAs on mRNA and protein expressions of EMT marker genes by RT-qPCR and Western blotting. GAPDH served as the endogenous control.

During cataract development, HLECs proliferate in the anterior capsule and gradually migrate to the posterior capsule, transforming from epithelial cells into a mesenchymal phenotype, which can be further transformed into myofibroblasts [17]. Moreover, a large amount of extracellular matrix components, such as collagen and fibronectin, is produced, ultimately leading to posterior capsular opacity and fibrosis [18]. Therefore, we investigated whether MALAT1 plays a role in modulating the proliferation, migration, and EMT of HLECs under inflammatory conditions. Wound healing and transwell assays showed that the migratory ability of HLECs was markedly improved under TNF-*α*-treated inflammatory conditions (Figures 5(a) and 5(b); Supplementary Material Figure S1). The results of the CCK8 experiment showed that TNF-*α* treatment notably improved the proliferation of HLECs (Figure 5(c)). Moreover, TNF-*α* treatment significantly increased the mRNA and protein levels of mesenchymal markers N-cadherin, *α*-SMA, vimentin, and Slug, whereas the expression of epithelial marker E-cadherin was decreased (Figures 5(d) and 5(e)). These results support the idea that the inflammatory environment could promote cataract development. However, interference with MALAT1 expression reversed the increased proliferative and migratory properties of HLECs that were mediated by the addition of TNF-*α* (Figures 5(a)–5(c)). In addition, the expression of E-cadherin was increased when MALAT1 was silenced; conversely, N-cadherin, *α*-SMA, vimentin, and Slug were suppressed (Figures 5(d) and 5(e)). These results demonstrate that interference with MALAT1 effectively inhibits the proliferation, migration, and EMT of HLECs under inflammatory conditions. In addition, we found that TNF-*α* aggravated the apoptosis of HLECs, while MALAT1 knockdown further increased the apoptosis rate (Supplementary Material Figure S2).

### 3.6. MALAT1 Negatively Regulates the Expression of miR-26a-5p

**Figure 6:**
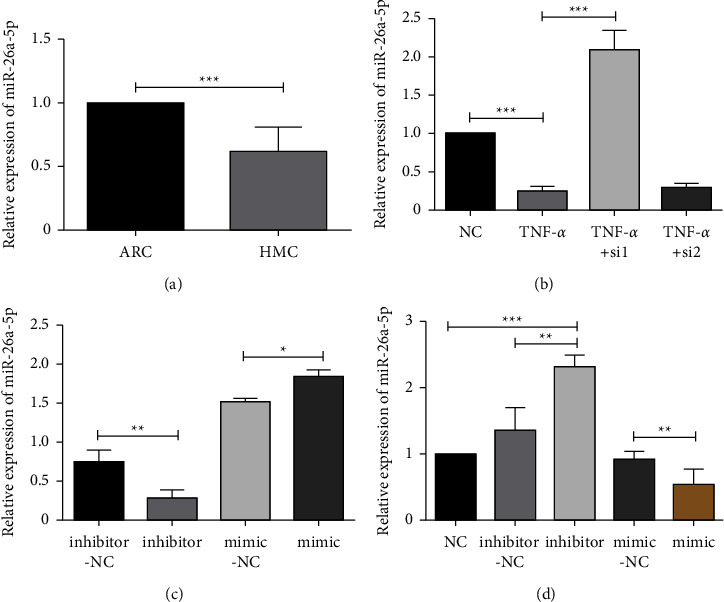
MALAT1 negatively regulates the expression of miR-26a-5p. (a) The expression level of miR-26a-5p in the capsular tissue of HMC patients was lower than that in the capsular tissue of ARC patients. (b) Evaluation of miR-26-5p expression in HLECs treated with TNF-*α* and MALAT1 siRNAs. (c) Evaluation of the changes in miR-26a-5p expression by RT-qPCR in HLECs transfected with miR-26a-5p inhibitors and mimics. (d) Evaluation of the changes in MALAT1 expression by RT-qPCR in HLECs transfected with miR-26a-5p inhibitors and mimics. GAPDH served as the endogenous control.

To explore the molecular mechanisms of MALAT1 for regulating the inflammatory response of HLECs, starBase and TargetScan were used to analyse the miRNAs that interact with MALAT1. We found that there was a potential combination between MALAT1 and miR-26a-5p (Supplementary Material Figure S3). It has been reported that MALAT1, acting as a ceRNA, regulates HMGB1 expression by binding to miR-26a-5p in saturated-fatty-acid-induced myocardial inflammatory injury, thereby inhibiting the activation of inflammatory responses [19]. Therefore, we hypothesised that miR-26a-5p may also be implicated in the MALAT1 signal that modulates the inflammatory response in HLECs. In this study, we found that the expression of miR-26a-5p in the capsular tissue of HMC patients was lower than that in ARC patients, which was opposite to the expression pattern of MALAT1 (Figure 6(a)). *In vitro*, miR-26a-5p expression was decreased by TNF-*α* treatment but increased by MALAT1 interference (Figure 6(b)). Moreover, miR-26a-5p mimics markedly suppressed the expression of MALAT1, whereas the inhibitors increased MALAT1 levels (Figures 6(c) and 6(d)). These results suggest that the expressions of MALAT1 and miR-26a-5p are inversely regulated *in vivo* and *in vitro* in HMC anterior capsular tissues and inflammatory-injured HLECs, respectively.

### 3.7. TET1 Is a Direct Downstream Target of miR-26a-5p

**Figure 7:**
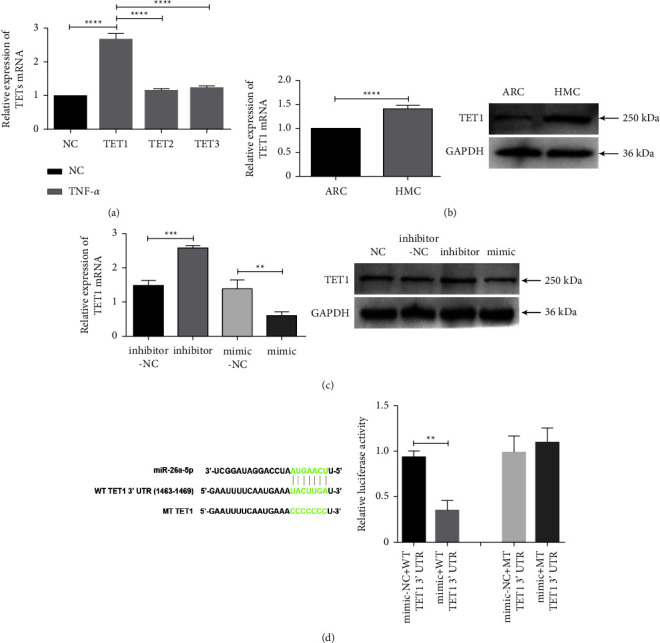
TET1 expression is regulated by miR-26a-5p. (a) Among the three TETs, TET1 expression was highest in TNF-*α*-treated HLECs. (b) TET1 expression increased in HMC tissues at the mRNA and protein levels. (c) Evaluation of the changes in TET1 mRNA and protein expression by RT-qPCR and Western blotting in HLECs transfected with miR-26a-5p inhibitors and mimics. (d) The luciferase assay identified TET1 as the direct target gene of miR-26a-5p.

To further investigate the mechanism underlying the modulation of the inflammatory response in HLECs, miRDB was used to determine the mRNAs involved in MALAT1/miR-26a-5p signaling. Members of the TET family were found to be the potential targets of miR-26a-5p. Among the three TETs, TET1 expression exhibited the most obvious change in the TNF-*α*-treated HLECs (Figure 7(a)). Several studies have shown that TET1 can modulate inflammatory conditions by regulating the expressions of IL-6, IL-8, TNF-*α*, and MCP-1 [20–22]. Four predicted miR-26a-5p binding sites were present in the 3′-UTR region of TET1 (Supplementary Material Figure S4), suggesting that miR-26a-5p can directly bind to TET1 mRNA and regulate the stabilisation of TET1 mRNA. We found that TET1 expression was increased in the HMC anterior capsular tissues at the mRNA and protein levels (Figure 7(b)), which is consistent with the expression pattern of MALAT1 but opposite to that of miR-26a-5p. After transfection with miR-26a-5p mimics and inhibitors, the mRNA and protein expressions of TET1 decreased and increased, respectively (Figure 7(c)). The luciferase assay results showed that miR-26a-5p was directly bound to TET1 mRNA (Figure 7(d)). Based on these data, we concluded that TET1 functions as a downstream target of miR-26a-5p in inflammation-damaged HLECs.

### 3.8. MALAT1 Interference Decreases TET1 Expression

**Figure 8:**
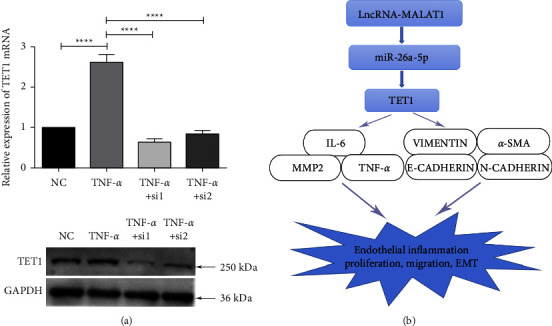
MALAT1 interference decreases TET1 expression. (a) Evaluation of the changes in TET1 mRNA and protein expressions by RT-qPCR and Western blotting in HLECs treated with TNF-*α* and MALAT1 siRNAs. GAPDH served as the endogenous control. (b) Diagram summarising the MALAT1/miR-26a-5p/TET1 signalling pathway in HLECs, which promotes the inflammatory response and EMT of HLECs.

To determine whether MALAT1 directly regulates the expression of TET1 under inflammatory conditions, we examined the expression of TET1 in TNF-*α*-treated and MALAT1-silenced HLECs. We showed that TET1 expression increased in TNF-*α*-treated HLECs but decreased in MALAT1-silenced HLECs. Notably, these changes are consistent with the variation in MALAT1 but in contrast to that in miR-26a-5p (Figure 8(a)). We speculated that MALAT1 stimulates the expression of TET1 through a competitive binding mechanism with miR-26a-5p and further promotes the inflammatory response, proliferation, migration, and EMT of HLECs, ultimately leading to HMC development (Figure 8(b)).

## 4. Discussion

A previous enzyme-linked immunosorbent assay revealed that the amounts of IL-6 and MMP-2 in the aqueous humour were significantly higher in highly myopic eyes, suggesting that intraocular inflammation may play an important role in the development and progression of high myopia and myopic retinopathy [23]. Previous studies and the data collected in the present study have shown that the expression levels of inflammatory factors IL-1, MMP-2, IL-10, TNF-*α*, MCP-1, IL-6, and NF-KB in the aqueous humour and anterior capsular tissue of HMC patients are higher than those of these factors in ARC patients [3, 4], indicating that the occurrence of this ophthalmic disease and its postoperative complications may be related to the inflammatory response. The level of IL-6 in the aqueous humour was higher in HMC patients than in ARC patients, and there was no significant difference in the serum levels between these two cases. Therefore, IL-6 in the aqueous humour of HMC eyes likely originates from the inflammatory microenvironment of the eye itself and is not leaked from the blood. This finding suggests that there is a proinflammatory environment in the eyes of HMC patients. In addition, there is a strong positive correlation between ocular axial length and the expression levels of IL-6 and MMP-2 in patients with high myopia. Interestingly, this correlation is stronger in men than in women [23].

TNF-*α* acts as a proinflammatory factor in the development of HMCs. TNF-*α* treatment induces the expression of several inflammatory proteins including IL, MCP, MMP, VEGF, and NF-KB. Importantly, these proteins form a positive feedback regulatory mechanism to enhance the expression of each other and then cooperatively promote the proliferation, migration, and EMT of HLECs [24]. In recent years, TNF-*α* has commonly been used to establish inflammatory injury cell models *in vitro* [25, 26], and it has been confirmed to regulate the transcription of MALAT1 through STAT3 and other pathways [11, 27, 28]. In this study, we treated HLECs with TNF-*α* to construct an inflammatory injury cell model for HMCs. We demonstrated that HLECs were transformed into spindle-shaped myofibroblasts after TNF-*α* treatment. MALAT1 expression was significantly upregulated in TNF-*α*-induced inflammatory HLECs, suggesting that inflammatory conditions could enhance the expression of MALAT1. In addition, TNF-*α* increased the proliferation, migration, and EMT of HLECs, while MALAT1 interference reversed these processes, suggesting that the inflammatory environment may change the biological behaviours of HLECs by regulating MALAT1 expression.

There is increasing evidence to suggest that lncRNAs, which are ceRNAs of miRNAs, are involved in the EMT process in inflammation-related ocular diseases [18, 29, 30]. We determined the expression pattern of lncRNAs in the anterior capsules of HMCs using whole transcriptome sequencing technology and identified MALAT1 as one of the lncRNAs with the most significant expression changes. MALAT1 was confirmed to participate in the progression of cancers through the regulatory expression of miRNAs [19, 31]. The lncRNA-MALAT1/miRNA-204-5p/Smad4 axis is involved in the regulation of the proliferation, migration, and EMT of HLECs [14]. Inspired by the ceRNA working mechanism, we determined miRNAs that bind to MALAT1 and mRNAs that serve as potential targets of select miRNAs. Two predicted binding sites exist between MALAT1 and miR-26a-5p, and four potential sites for interaction between miR-26a-5p and TET1 mRNA were identified. Although a regulatory relationship between MALAT1 and miR-26a-5p has been reported [32], the molecular connection between miR-26a-5p and TET1 and between MALAT1 and TET1 has not been studied. In our *in vivo* and *in vitro* data, the expression trend of miR-26a-5p was opposite to that of MALAT1, whereas TET1 expression was consistent with MALAT1 expression. MALAT1 interference markedly decreased TET1 expression. In addition, as miR-26a-5p directly binds to TET1, treatment with miR-26a-5p mimics and inhibitors suppressed or enhanced the expression of MALAT1 and TET1. Thus, our findings suggest that MALAT1 could act as a ceRNA of miR-26a-5p to regulate the expression of TET1. The lncRNA-MALAT1/miR-26a-5p/TET1 signalling axis is involved in regulating the inflammatory response, proliferation, migration, and EMT of HLECs, and it may serve as a target for the clinical treatment of HMCs.

## 5. Conclusions

HMCs are an intraocular inflammatory stress-related disease. lncRNA-MALAT1 is highly expressed in the precapsular tissue of HMCs, and its expression is associated with inflammatory states. MALAT1 acts as a ceRNA of miR-26a-5p to regulate the expression of TET1, and this signalling is responsible for the inflammatory response, proliferation, migration, and EMT of inflammatory HLECs.

## Data Availability

Data are available upon request to the corresponding author.

## Supplementary Materials

Supplementary Materials Figure S1 represents the statistical diagram of migrating cells and the wound healing area. Figure S2 represents the apoptosis proportion of HLECs treated with TNF-*α* and MALAT1 siRNAs. Figure S3 shows the predicted binding sites between MALAT1 and miR-26a-5p. Figure S4 shows the predicted binding sites between miR-26a-5p and TET1. Table S1 lists the sequence information of MALAT1 siRNAs. Table S2 lists the sequence information of miR-26a-5p mimics and inhibitors. Table S3 shows the primers used for real-time qPCR. Table S4 shows the antibodies used for the Western blot.
