# Supplementary material for: Regulation of the Inflammatory Response, Proliferation, Migration, and Epithelial-Mesenchymal Transition of Human Lens Epithelial Cells by the lncRNA-MALAT1/miR-26a-5p/TET1 Signaling Axis
Source: J Ophthalmol. 2023 Jan 16;2023:9942880. doi: 10.1155/2023/9942880 (PMC9870684; doi:10.1155/2023/9942880)
Supplement: Supplementary Materials — Figure S1 represents the statistical diagram of migrating cells and the wound healing area. Figure S2 represents the apoptosis proportion of HLECs treated with TNF-α and MALAT1 siRNAs. Figure S3 shows the predicted binding sites between MALAT1 and miR-26a-5p. Figure S4 shows the predicted binding sites between miR-26a-5p and TET1. Table S1 lists the sequence information of MALAT1 siRNAs. Table S2 lists the sequence information of miR-26a-5p mimics and inhibitors. Table S3 shows the primers used for real-time qPCR. Table S4 shows the antibodies used for the Western blot. [file 9942880.f1.docx]

Supplementary Material

**Regulation of the inflammatory response, proliferation, migration, and epithelial–mesenchymal transition of human lens epithelial cells by the LncRNA-MALAT1/miR-26a-5p/TET1 signalling axis**

Yaru Hu^1^, Xue Han^1^, Yue Chen^1^, Jinbiao Cai^1^, Juan Li^1^, Yuchen Fan^1^, Jianfeng Wang^1*^, Shanglun Xie^2*^

^1^Department of Ophthalmology, The First Affiliated Hospital of Bengbu Medical College, Bengbu, Anhui; 233004, China

^2^School of Life Sciences, Anhui Province Key Laboratory of Translational Cancer Research, Bengbu Medical College, Anhui, 233030, China

^*^ Corresponding author

1. mail address: [shanglunxie@bbmc.edu.cn](mailto:shanglunxie@bbmc.edu.cn)

**Figure S1-S4**


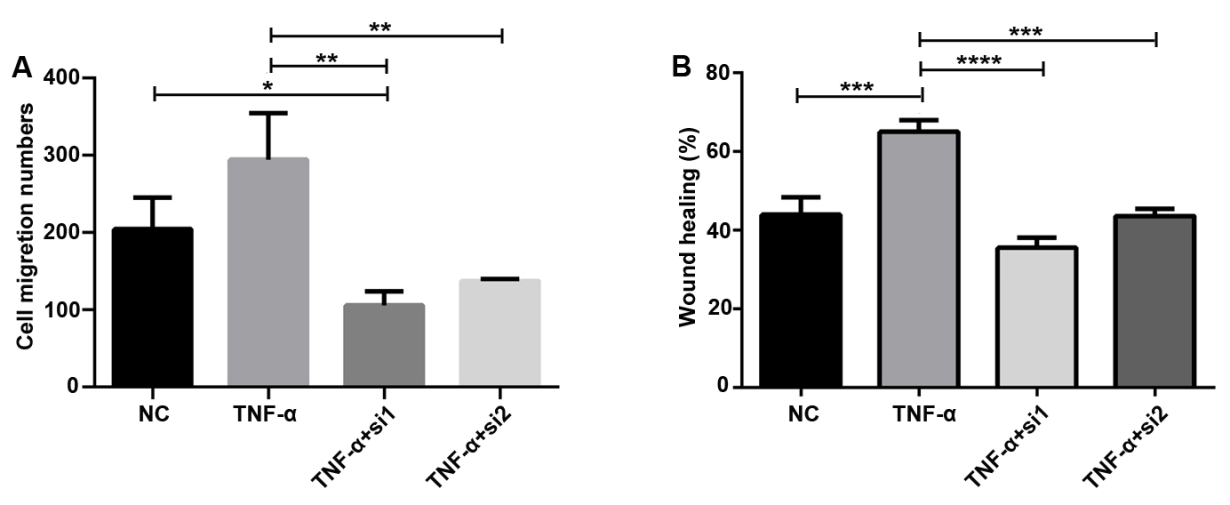


**Figure S1**: The statistical diagram of migrating cells (A) and wound healing area (B) in Figure 5A and 5B.

**
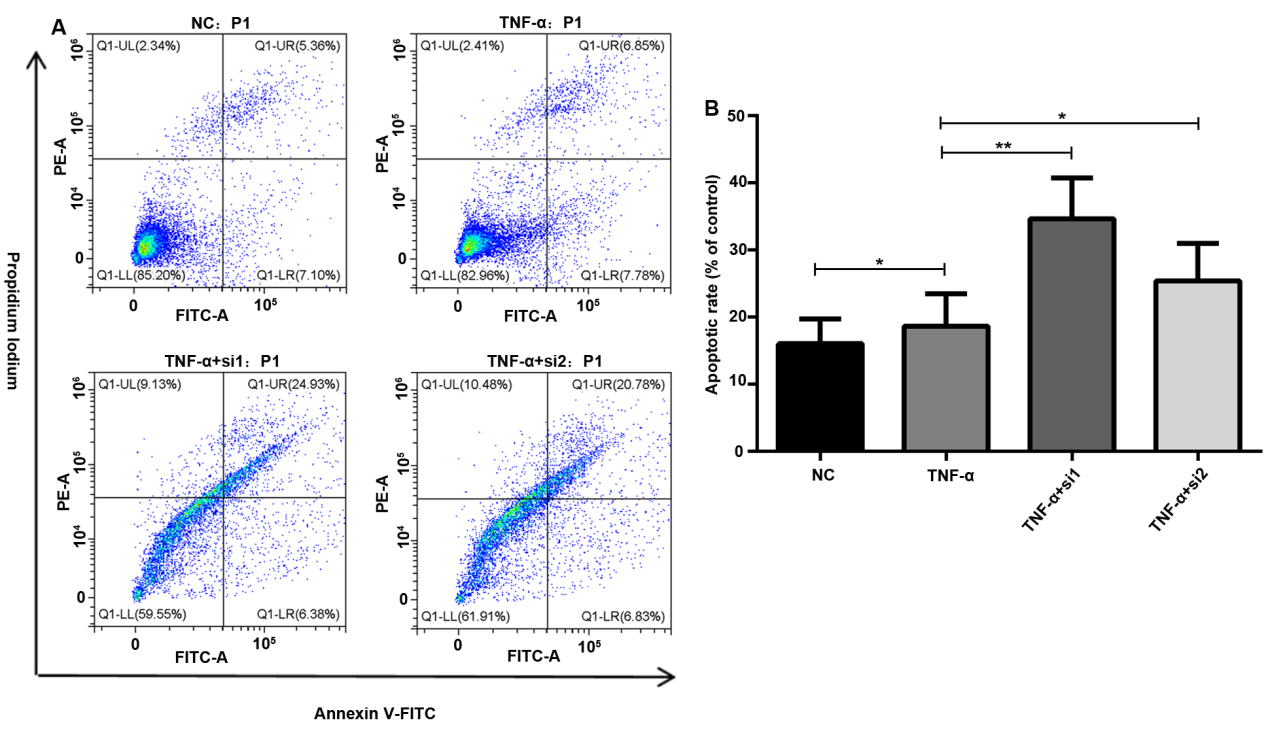
**

**Figure S2**: (A) Flow cytometry analyzed the apoptosis proportion of HLECs treated with TNF-α and MALAT1 siRNAs . (B) The statistical diagram of the apoptotic rate.

**
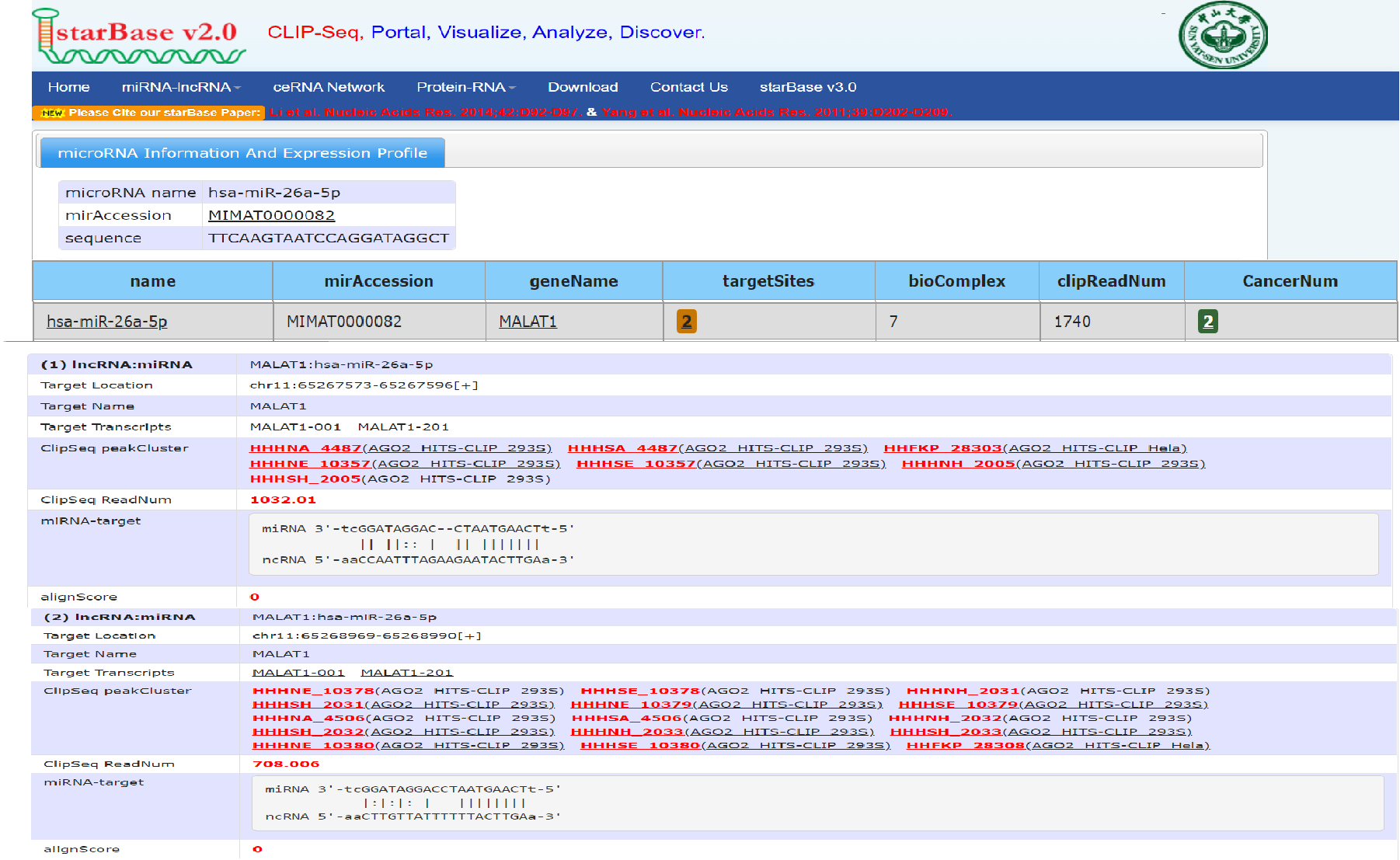
Figure S3**: Starbase v 2.0 (http: //starbase. sysu.edu.cn) was used to predict the binding sites between MALAT1 and miR-26a-5p.


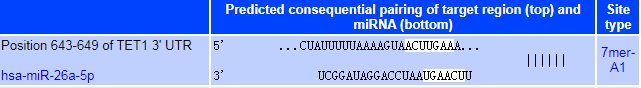

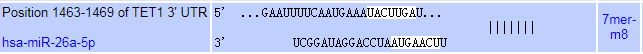

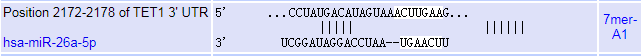

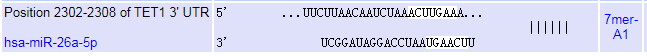


**Figure S4:** Lianchuan bioinformatics analysis predict the binding sites between miR-26a-5p and the 3’UTR of TET1.

**Table S1-S4**

**Table S1**. The sequences are used to construct MALAT1 siRNA

|  | **Sense (5’ to 3’)** | **Antisense (5’ to 3’)** |
| --- | --- | --- |
| siRNA 1 | GGCAAUGUUUUACACUAUUTT | AAUAGUGUAAAACAUUGCCTA |
| siRNA 2 | UUCUCCGAACGUGUCACGUTT | ACGUGACACGUUCGGAGAATT |

**Table S2**. The sequences are used to construct miR-26a-5p mimics and inhibitors

|  | **Sense (5’ to 3’)** | **Antisense (5’ to 3’)** |
| --- | --- | --- |
| mimics | UUCAAGUAAUCCAGGAUAGGCU | AGCCUAUCCUGGAUUACUUGAA |
| inhibitors | AGCCUAUCCUGGAUUACUUGAA | UUCAAGUAAUCCAGGAUAGGCU |

**Table S3**. Primers used for real-time qPCR

| **Gene names** | **Primer sequences (5’ to 3’)** |
| --- | --- |
| MALAT1 F | AAGAACTGTAATGCTGGGTGGGAAC |
| MALAT1 R | AAGACGCCGCAGGGATTTGAAC |
| miR-26a-5p F | GCTCTGAACGTAGATCCGAAC |
| miR-26a-5p R | GTGCAGGGTCCGAGGT |
| TET1 F | AGAAAAGGGAAAGGAGAGCG |
| TET1 R | TCTTCCCCATGACCACATCT |
| TNF-α F | CACTGCCACCAAGAGCCAAGAG |
| TNF-α R | CCCATTAGAGTGCCTTACCGTTGAC |
| IL-6 F | TGAGAGTAGTGAGGAACAAGCCAGAG |
| IL-6 R | GAAGAGAGCCAACCAACCAAACAAAC |
| MMP-2 F | AGTCCTACCAACCTCCTGTCTCATTC |
| MMP-2 R | CCAAGCGGTCTAAGTCCAGAGTTATC |
| MMP-9 F | TCCTCTTATGCCTGCCTGTCTCC |
| MMP-9 R | CCACCTCCACTCCTCCCTTTCC |
| GAPDH F | CTGGGCTACACTGAGCACC |
| GAPDH R | AAGTGGTCGTTGAGGGCAATG |
| N-cadherin F | CCTCCAGAGTTTACTGCCATGAC |
| N-cadherin R | GTAGGATCTCCGCCACTGATTC |
| E-cadherin F | GGCGCCACCTGGAGAGA |
| E-cadherin R | TGTCGACCGGTGCAATCTT |
| α-SMA F | CTGAACCCCAAGGCCAACCG |
| α-SMA R | GACAATCTCACGCTCAGCAGT |
| Vimentin F | TACAGGAAGCTGCTGGAAGG |
| Vimentin R | ACCAGAGGGAGTGAATCCAG |

F: Forward primer sequence

R: Reverse primer sequence

**Table S4**. Antibodies used for western blotting

| **Protein name** | **Antibodies** |
| --- | --- |
| TET1 | A6422, AB clonal |
| IL6 | [DF6](http://www.affbiotech.com/goods-18140-BF8006-Vimentin_mouse_monoclonal_Antibody.html)087, affinity |
| MMP-2 | [AF0](http://www.affbiotech.com/goods-18140-BF8006-Vimentin_mouse_monoclonal_Antibody.html)577, affinity |
| E-cadherin | 20874-1-AP, proteintech |
| N-cadherin | 22018-1-AP, proteintech |
| α-SMA | 14395-1-AP, proteintech |
| VIMENTIN | 5741, CST |
| SLUG | 9585, CST |
| GAPDH | 60004-1-Ig, proteintech |
